# Compound Danshen Dripping Pill Inhibits Retina Cell Apoptosis in Diabetic Rats

**DOI:** 10.3389/fphys.2018.01501

**Published:** 2018-10-24

**Authors:** Qian Zhang, Xinhua Xiao, Jia Zheng, Ming Li, Miao Yu, Fan Ping, Tong Wang, Xiaojing Wang

**Affiliations:** Key Laboratory of Endocrinology, Translational Medicine Center, Ministry of Health, Department of Endocrinology, Peking Union Medical College Hospital, Peking Union Medical College, Chinese Academy of Medical Sciences, Beijing, China

**Keywords:** diabetic retinopathy, Danshen, apoptosis, retina, neuropeptide Y

## Abstract

**Scope:** Diabetic retinopathy (DR) is a severe microvascular complication of diabetes. Previous clinical trials have shown that Compound Danshen Dripping Pill (CDDP) improves DR symptoms. However, the mechanism involved remains unclear.

**Procedures:** Rats fed a high-fat diet and injected with streptozotocin (STZ) were used as an experimental type 2 diabetes rodent model. CDDP was administered to two groups of diabetic rats at 0.2 and 0.4 g/kg/day via gastric gavage for 12 weeks. After the 12 weeks of treatment, retinal function was evaluated by electroretinography (ERG). Histological staining and TdT-mediated dUTP nick-end labeling (TUNEL) assays were also performed. Retinal genome expression was determined by gene array.

**Results:** We found that CDDP moderated ERG and histological abnormalities in diabetic rats, independent of blood glucose level. A gene array showed that CDDP changed 262 genes significantly in the diabetic retina. Kyoto Encyclopedia of Genes and Genomes (KEGG) pathway analysis indicated that differentially expressed genes in the CDDP-treated groups were involved mainly in the apoptosis pathway. Moreover, CDDP reduced the number of TUNEL-positive cells in the diabetic retinas. CDDP prevented the reduction in Bcl-2 expression and the increase in BCL-2 associated X (Bax) and caspase-3 (Casp3) expression in diabetic rats.

**Conclusion:** Our results suggest that CDDP exerts its neuroprotective functions by inhibiting cell apoptosis in diabetic rats.

## Introduction

Diabetic retinopathy (DR) is one of the main complications of diabetes (Cheung et al., 2010). It is the leading cause of blindness in patients aged 20–70 years. DR has become an important public health problem that affects more than 90% of diabetic patients. However, the current treatment for DR is for only advanced stages of the disease (Simo and Hernandez, 2009). Drugs for early stage intervention in DR are limited.

Previously, scientists believed that DR was only a diabetic microvascular complication (Abu-El-Asrar et al., 2004). However, increasing evidence shows that all retinal cells possess pathological changes from the early stages of diabetes (Lieth et al., 2000; Gardner et al., 2002). Emerging evidence shows that neurodegeneration of the retinal neurodegeneration occurs before vascular lesions in patients with diabetes and becomes an early pathogenic event in DR (Barber et al., 2011). Retinal neurodegeneration is an important event that induces microcirculatory abnormalities from the early stages of DR (Antonetti et al., 2012).

Danshen (*Salvia miltiorrhiza*), a Chinese herb, can be used to treat blood stasis and improve blood circulation. In fact, Danshen has been used widely for treating angina pectoris, myocardial infarction, and stroke. Compound Danshen Dripping Pill (CDDP) is the first traditional Chinese drug that has been approved by the American FDA in phase II clinical trials for the treatment of cardiovascular disease (Luo et al., 2013). The results of two clinical trials reveal that CDDP improves the symptoms of DR (Lian et al., 2015; Luo et al., 2015). Animal experiments show that Danshen prevents oxidative stress in the eyes of diabetic rats (Yue et al., 2006).

However, the exact mechanism of CDDP protection against DR remains unknown, particularly its neuroprotective mechanism. Our study was designed to utilize a whole transcriptome expression array to determine the important changes in gene expression that occur in the genomic profiles of early DR rats treated with CDDP. These results will help determine the potential protective mechanism of CDDP in patients with DR.

## Materials and Methods

### Study Design and Animal Experiments

Five-week-old male SD rats (132.9 ± 10.3 g) were purchased from the Institute of Laboratory Animal Science, Chinese Academy of Medical Sciences and Peking Union Medical College (Beijing, China, SCXK-2014-0013). All rats were maintained in cages at 24 ± 1°C with the lights on from 6:00 a.m. to 6:00 p.m.

Control rats (*n* = 6) were fed a standard diet (kcal%: 10% fat, 20% protein, and 70% carbohydrate; 3.85 kcal/gm). Other rats (*n* = 18) were fed a high fat diet (kcal%: 45% fat, 20% protein, and 35% carbohydrate; 4.73 kcal/gm, Research Diet, New Brunswick, NJ, United States) for 4 weeks and were then injected intraperitoneally with streptozotocin (STZ, 30 mg/kg body weight) to induce diabetes (Guo et al., 2011; Ren et al., 2012; Davidson et al., 2014; Skovso, 2014; Zhang et al., 2016). Fasting blood glucose levels were measured with a Bayer Contour TS glucometer (Bayer, Hamburg, Germany). Fasting blood glucose (FBG) levels >11.1 mmol/L were used to indicate the successful establishment of the type 2 diabetes mellitus (T2DM) model. Then, the diabetic rats were divided randomly into the following three groups: diabetic group (*n* = 6), low dose CDDP group (LCDDP, *n* = 6, 0.2 g/kg/d), and high dose CDDP (HCDDP, *n* = 6, 0.4 g/kg/d) group. CDDP was prepared by Tasly Pharmaceutical Group Co., Ltd. (Tianjin, China) and included Danshen (*S. miltiorrhiza*), notoginseng (*Panax notoginseng*) and borneol. CDDP was dosed via gastric gavage once daily for 12 weeks in the LCDDP group and HCDDP groups. The control and diabetic groups received 0.5% saline. After 12 weeks of treatment, the rats were sacrificed by cervical dislocation. The fresh eyeballs were immediately enucleated. The retinas were separated and stored at -80°C to assess of RNA expression. The other eye was fixed in 4% paraformaldehyde for 24 h.

### Electroretinography (ERG)

Electroretinography activity was assessed after the 12 weeks of treatment. Before the ERG tests, the rats were dark-adapted for 12 h and anesthetized intraperitoneally with 2% pentobarbital (50 mg/kg) under a dim red light. The pupils were fully dilated with topical 1% tropicamide (Santen Pharmaceutical Co., Ltd., Japan). A Ganzfeld stimulator with a flash intensity of 3 cd.s.m^-2^ generated light stimuli for the rats. Corneal electrodes recorded the flash ERG responses for both eyes. The negative and ground electrodes were placed in the subcutaneous space of the cheek and the tail, respectively. ERG signals were collected by an ESPION Console (Diagnosys LLC, Littleton, MA, United States). The ERG signals were amplified 20,000-fold, and filtered for 10–300 Hz using an amplifier. The amplitude of the ERG waves was measured as the difference between the maximum positive (stimulation) and negative (baseline) peaks. The oscillatory potentials (OPs) were measured as four to six wavelets from the rising phase of the b-wave. The magnitude of the OPs was calculated as the sum of the three major amplitudes.

### Eyeball Histological Analysis

The eyeballs fixed in 4% paraformaldehyde were then cryoprotected in 30% sucrose, dehydrated in alcohol, and embedded in paraffin. Sections (5 μm) were stained with hematoxylin and eosin (H&E). Digital images were obtained using a Nikon Eclipse 90i digital microscope with the NIS-Elements 3.10 Image Analysis System (Nikon Instruments Inc., Lewisville, TX, United States). The total retinal thickness, inner nuclear layer (INL) thickness and outer nuclear layer (ONL) thickness were measured at 400× magnification. Two measurements were taken for each section at the two reference lines, which were 1 mm away from the optic nerve on both the superior and inferior sides. Cell numbers in the INL and the ONL were counted in the same region at 1000× magnification. All the cell nuclei within a fixed 25-μm column and centered with the 1-mm reference lines, were counted. The results from five sections per eye were averaged and there were six rats in each group.

### RNA Isolation and Gene Expression Array

Total RNA from the rat retinas was extracted using a mirVana^TM^ RNA Isolation Kit (Ambion, São Paulo, Brazil). Double-stranded cDNA was synthesized from RNA. Then cDNA was labeled with biotin. The biotinylated cDNA was purified, fragmented, and hybridized to an Affymetrix GeneChip Rat Gene 2.0 ST whole transcript-based array (Affymetrix Technologies, Santa Clara, CA, United States). After washing and staining, the microarrays were scanned using an Affymetrix Scanner 3000 7G (Santa Clara, CA, United States). Microarray signals were analyzed using Expression Console Software 1.41 (Affymetrix, Santa Clara, CA, United States). The final gene list contained only those proven sets with a *P* < 0.05 and fold change >1.5. For the interpretation and visualization of the data, Database for Annotation, Visualization, and Integrated Discovery (DAVID^1^) and the Kyoto Encyclopedia of Genes and Genomes (KEGG) database were used. The array data have been submitted to the GEO database (GSE115866^2^, for reviewers only).

### Quantitative Real-Time PCR

Total RNA from the retinas was used to synthesize cDNA with SuperScript II reverse transcriptase (Life Technologies, Carlsbad, CA, United States). Quantitative PCR was performed using gene-specific primers (Table 1), SYBR Green and an ABI Prism 7500 Real-Time System (Applied Biosystems, Foster City, CA, United States). Relative expression levels were calculated with the 2^-ΔΔCt^ method.

**Table 1:** Primer sequences for quantitative real-time PCR analysis.

Gene symbol	Gene bank ID	Forward primer	Reverse primer	Product size (bp)
Casp3	NM_012922	GGAGCTTGGAACGCGAAGAA	ACACAAGCCCATTTCAGGGT	169
Bcl-2	NM_016993	CTGGTGGACAACATCGCTCT	GCATGCTGGGGCCATATAGT	115
TIMP1	NM_053819	TGCTCAAAGGATTCGACGCT	AGCAGGGCTCAGATTATGCC	200
Bax	NM_017059	AGGACGCATCCACCAAGAAG	CAGTTGAAGTTGCCGTCTGC	166
NPY	NM_012614	TGGCCAGATACTACTCCGCT	TTCAAGCCTTGTTCTGGGGG	143

*Casp3, Caspase 3; Bax, BCL2 associated X; Npy, neuropeptide Y.*

### Immunohistochemistry for Bcl-2 and Bcl-2 Associated X (Bax) in the Retina

Paraffin-embedded retina sections were dewaxed and rehydrated. The sections were washed with phosphate-buffered saline (PBS), incubated with 10% goat serum in PBS for 30 min at room temperature, and incubated with an anti-Bcl-2 antibody (Cat. No. sc-7382, 1:200, Santa Cruz Biotechnology, Dallas, TX, United States) or anti-Bax antibody (Cat. No. sc-7480, 1:200, Santa Cruz Biotechnology, Dallas, TX, United States) at 4°C overnight. The next day, the sections were washed with PBS three times for 5 min and incubated with an horseradish peroxidase (HRP)-conjugated secondary antibody (Cat. No. sc-516102, 1:2000, Santa Cruz Biotechnology, Dallas, TX, United States) for 50 min at room temperature. After washing with PBS, the sections were stained with a 3,3′-diaminobenzidine (DAB) Color Development Kit (ZSGB-BIO, Beijing, China). Hematoxylin was used to stain the nuclei. The slides were analyzed using ImageJ Software (National Institutes of Health, Baltimore, MD, United States) at 400× magnification. Positive cells were brownish yellow. Five slides rat were analyzed, and there were with six rats per group.

### Apoptosis Assessment in the Retina

Apoptosis was evaluated with the TdT-mediated dUTP nick-end labeling (TUNEL) method. Briefly, paraffin-embedded sections were dewaxed, washed with PBS and incubated with Proteinase K for 20 min. After washing with PBS, 50 μL of reaction solution (TUNEL Apoptosis Detection Kit, Roche Applied Science, Mannheim, Germany) was added to each slide at 37°C for 60 min in the dark. After washing, the sections were incubated with 3% hydrogen peroxide for 20 min. Then, 50 μL of Converter-POD (TUNEL Apoptosis Detection Kit, Roche Applied Science, Mannheim, Germany) was added to the sections at 37°C for 30 min. Finally, a DAB Color Development Kit (ZSGB-BIO, Beijing, China) was used to stain the slides. Hematoxylin was used to stain the nuclei. The slides were analyzed using ImageJ Software (National Institutes of Health, Baltimore, MD, United States) at 400× magnification.

### Data Analysis

The values are expressed as the mean ± SD. Differences among the groups were compared with one-way ANOVA followed by Turkey’s *post hoc* test. Differences between two groups were compared with unpaired Student’s *t*-test. For gene ontology (GO) and KEGG pathway analyses, Fisher’s exact test was used. Differences were considered statistically significant for *P*-values <0.05. GraphPad Prism Software 5.0 (San Diego, CA, United States) was used for data analysis.

### Ethics Statement

Procedures involving animals and their care were conducted in accordance with international and national law and policies (EU Directive 2010/63/EU for animal experiments, ARRIVE guidelines and the Basel Declaration including the 3R concept). All procedures were performed with the approval of the Animal Care Committee of the Peking Union Medical Hospital Animal Ethics Committee (Project XHDW-2015-0051, February 15, 2015), and all efforts were made to minimize animal suffering.

## Results

### The Effect of CDDP on Body Weight and FBG Levels in Diabetic Rats

**FIGURE 1:**
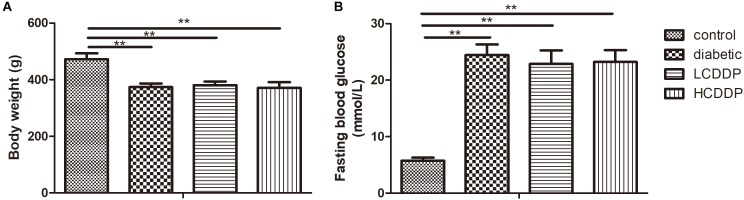
The effect of CDDP on body weight **(A)** and blood glucose levels **(B)** in diabetic rats. LCDDP, low dose Compound Danshen Dripping Pill; HCDDP, high dose Compound Danshen Dripping Pill. *n* = 6 in each group. ^∗∗^*P* < 0.01.

The body weight was lower in the diabetic group than in the control group (*P* < 0.01, Figure 1A). The CDDP groups also had comparable body weight to those of the diabetic group (*P* > 0.05, Figure 1A). The diabetic group had higher plasma glucose levels than the control group (*P* < 0.01, Figure 1B). Neither the low dose nor the high dose of CDDP decreased blood glucose levels in the diabetic rats (*P* > 0.05, Figure 1B).

### The Effect of CDDP on the ERG Results in Diabetic Rats

**FIGURE 2:**
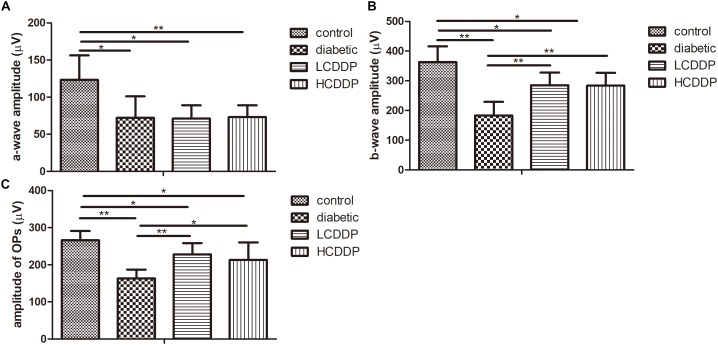
The effect of CDDP on the amplitude of the a-waves **(A)**, b-waves **(B)**, and OPs **(C)** in diabetic rats. LCDDP, low dose Compound Danshen Dripping Pill; HCDDP, high dose Compound Danshen Dripping Pill. *n* = 6 in each group. ^∗^*P* < 0.05, ^∗∗^*P* < 0.01.

To evaluate the effect of CDDP on DR, full-field flash ERG was measured. The amplitudes of the a-waves (*P* < 0.01, Figure 2A), b-waves (*P* < 0.01, Figure 2B), and OPs were significantly reduced in diabetic rats (*P* < 0.01, Figure 2C). Although CDDP did not moderate the amplitudes of the a-waves in diabetic rats (*P* > 0.05, Figure 2A), it enhanced the amplitudes of the b-waves and OPs (*P* < 0.05, Figures 2B,C). These results indicate that CDDP restored retina function in diabetic rats.

### The Effect of CDDP on Retinal Morphometry

**FIGURE 3:**
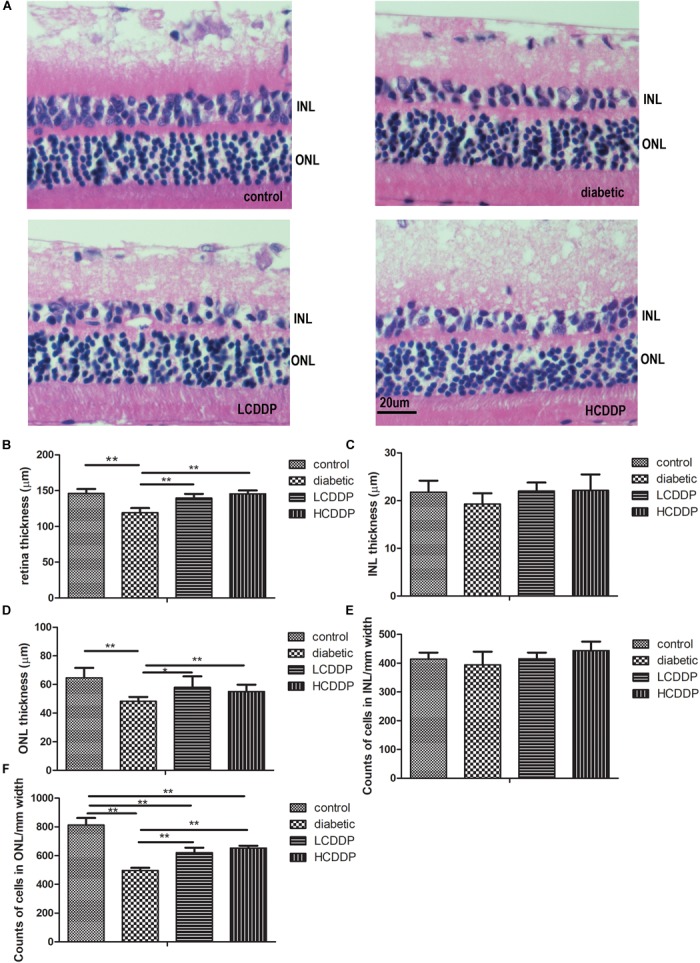
The effect of CDDP on retina morphometry in diabetic rats. **(A)** H&E stained sections (400×), **(B)** total retinal thickness, **(C)** INL thickness, **(D)** ONL thickness, **(E)** cell counts in the INL, **(F)** cell counts in the ONL. The total retinal thickness, INL thickness and ONL thickness were measured at 400× magnification. Two measurements were taken from each section, at the two reference lines, which were 1 mm away from the optic nerve on both the superior and inferior sides. Cell numbers in the INL and ONL were counted in the same region at 1000× magnification. All the cell nuclei within a fixed 25-μm column and centered with the 1-mm reference lines, were counted. The results from five sections per eye were averaged. LCDDP, low dose Compound Danshen Dripping Pill; HCDDP, high dose of Compound Danshen Dripping pill; INL, inner nuclear layer; ONL, outer nuclear layer. The results from five sections per eye were averaged. *n* = 6 in each group. ^∗^*P* < 0.05, ^∗∗^*P* < 0.01.

The total retina thickness and the ONL thickness were lower in the diabetic rats than in the control rats (*P* < 0.01, Figures 3A,B,D). The cell count in the ONL was also lower in diabetic rats than in the control rats (*P* < 0.01, Figures 3A,F). CDDP increased the total retina thickness, ONL thickness and cell counts in the ONL (*P* < 0.05, Figures 3A,B,D,F). There were no significant change among the four groups in INL thickness or the INL cell counts (*P* > 0.05, Figures 3A,C,E).

### Gene Array Analysis

**FIGURE 4:**
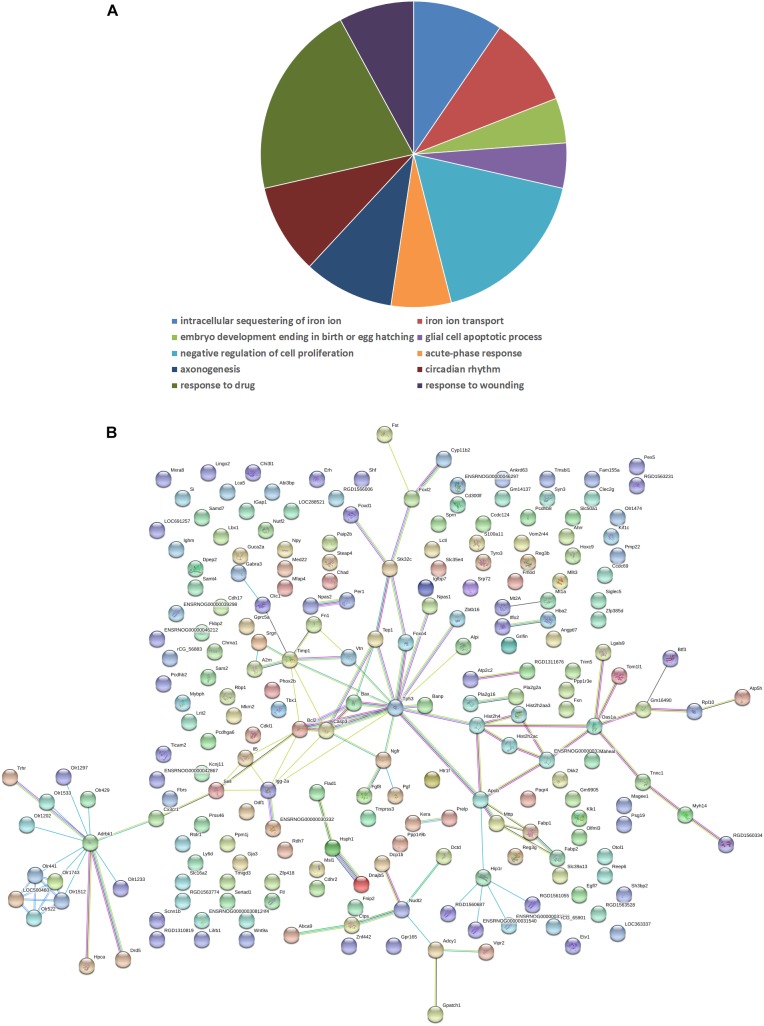
Biology process **(A)** and interaction network **(B)** in differentially expressed genes between the HCDDP and diabetic groups. The nodes represent differentially expressed genes. The lines represent the interactions between genes.

To reveal the molecular mechanisms underlying in the retinal effects CDDP in diabetic rats, we performed a genome-wide expression profiling analysis on total RNA isolated from the retinas of the HCDDP group and the diabetic group (*n* = 3 in each group) (Hargadon, 2015; Funderburk et al., 2017; Gambara et al., 2017; Li et al., 2017). We found that 262 genes were differentially expressed in the retinas of HCDDP-treated rats (fold change >1.5, *P* < 0.05). Of these genes, 116 genes were upregulated, 146 genes were downregulated. The differentially expressed genes were enriched in six pathways (*P* < 0.05, Table 2). The top five pathways of these genes were fat digestion and absorption, amyotrophic lateral sclerosis, pathway in cancer, apoptosis, and colorectal cancer. The top ten genes were related to significant biology processes (BPs) in the GO categories, including intracellular sequestering of iron ion, iron ion transport, embryo development, ending in birth, or egg hatching, negative regulation of cell proliferation, acute-phase response, axonogenesis, circadian rhythm, response to drug, and response to wounding (*P* < 0.01, Figure 4A and Table 3). String interaction analysis showed twelve genes that had more than five interactions with other differentially expressed genes, including Tp53 (tumor protein p53), Adrbk1 (G protein-coupled receptor kinase 2), Timp1 (TIMP metallopeptidase inhibitor 1), Bcl-2, Caspase 3 (Casp3), Apob (apolipoprotein B), IgG-2a (gamma-2a immunoglobulin heavy chain), Hip1r (huntingtin interacting protein 1 related), Hist2h4 (histone cluster 2 H4), Olr1512 (olfactory receptor 1512), Olr1743 (olfactory receptor 1743), and Olr441 (olfactory receptor 441, Figure 4B).

**Table 2:** The enriched KEGG pathway with differentially expressed genes (*P* < 0.05).

Pathway ID	Pathway name	Count	Genes	Fold enrichment	*P*-value
rno04975	Fat digestion and absorption	5	APOB, PLA2G2A, FABP1, FABP2, MTTP	12.33354471	0.000644
rno05014	Amyotrophic lateral sclerosis (ALS)	4	CASP3, BCL2, BAX, TP53	6.817086528	0.0200
rno05200	Pathways in cancer	10	ADCY1, FGF8, CASP3, PGF, BCL2, BAX, TP53, WNT9A, ZBTB16, FN1	2.35514924	0.0236
rno04210	Apoptosis	4	CASP3, BCL2, BAX, TP53	6.047415468	0.0273
rno05210	Colorectal cancer	4	CASP3, BCL2, BAX, TP53	5.858433735	0.0297
rno05034	Alcoholism	6	HIST1H4M, HIST2H2AA3, NPY, HIST2H2AC, LOC103690190, HIST2H4	3.19550931	0.0378

**Table 3:** The enriched GO terms with differentially expressed genes (*P* < 0.01).

Term ID	Term name	Count	*P*-value	Genes	Fold enrichment	Category
0006880	Intracellular sequestering of iron ion	6	8.06E-07	FTL1L1, LOC100359668, LOC100360087, FTL1, RGD1560687, LOC100362384	32.47222	Biological process
0006826	Iron ion transport	6	1.44E-05	FTL1L1, LOC100359668, LOC100360087, FTL1, RGD1560687, LOC100362384	18.85484	Biological process
0009792	Embryo development ending in birth or egg hatching	3	0.002787	FGF8, FXN, TP53	36.53125	Biological process
0034349	Glial cell apoptotic process	3	0.004419	CASP3, BCL2, BAX	29.225	Biological process
0008285	Negative regulation of cell proliferation	11	0.005164	PHOX2B, LBX1, BCL2, BAX, TP53, PLA2G2A, LOC103690190, WNT9A, ZBTB16, FOXO4, PMP22	2.865196	Biological process
0006953	Acute-phase response	4	0.006789	REG3B, A2M, REG3G, FN1	10.25439	Biological process
0007409	Axonogenesis	6	0.007003	LINGO2, FMOD, ADCY1, KERA, BCL2, PRELP	4.995726	Biological process
0007623	Circadian rhythm	6	0.008044	ADCY1, NPAS2, TP53, PER1, NGFR, MTTP	4.830579	Biological process
0042493	Response to drug	13	0.008112	PPP1R9B, ADCY1, FGF8, CASP3, FXN, PGF, BCL2, BAX, TP53, SRP72, KCNJ11, HTR1F, FN1	2.398516	Biological process
0009611	Response to wounding	5	0.009094	CASP3, A2M, BAX, NGFR, FN1	6.088542	Biological process
0042060	Wound healing	6	0.009491	FMOD, CASP3, DRD5, TP53, TIMP1, FN1	4.638889	Biological process
0072562	Blood microparticle	8	1.90E-04	IGHG, A2M, IGG-2A, VTN, HBA2, CLIC1, LOC100361105, FN1	6.714402	Cellular component
0031012	Extracellular matrix	11	2.55E-04	FMOD, HIST1H4M, EGFL7, IGFBP7, VTN, MFAP4, ABI3BP, PRELP, TIMP1, HIST2H4, FN1	4.289023	Cellular component
0005615	Extracellular space	27	7.06E-04	FMOD, A2M, FGF8, KERA, PGF, IGFBP7, VTN, FBRS, ABI3BP, TIMP1, REG3B, APOB, SRGN, FN1, IL5, EGFL7, RGD1563231, S100A11, CHI3L1, CLIC1, LGALS9, PRELP, DKK2, IGHG, NPY, PLA2G2A, WNT9A	2.031923	Cellular component
0070062	Extracellular exosome	42	0.0027	STEAP4, HIST2H2AA3, HIST1H4M, ADCY1, A2M, FTL1, IGFBP7, VTN, GPRC5A, TIMP1, RGD1560334, HSPH1, APOB, HIST2H2AC, LOC103690190, GUCA2A, SCNN1B, ATP5H, FN1, MLLT3, DCTD, SI, CDHR2, S100A11, CHI3L1, HBA2, MXRA8, CLIC1, LGALS9, HIST2H4, PRELP, IGHG, CDKL1, TOM1L1, BAX, PLA2G2A, NUTF2, FABP1, MYH14, WNT9A, MFAP4, FKBP2	1.572023	Cellular component
0005578	Proteinaceous extracellular matrix	9	0.004099	FMOD, KERA, VTN, WNT9A, ABI3BP, PRELP, TIMP1, CHAD, FN1	3.523071	Cellular component
0005783	Endoplasmic reticulum	17	0.008741	PLA2G16, SLC39A13, TP53, CHI3L1, KCNJ11, TMPRSS3, MTTP, RDH7, APOB, LCTL, BAX, BCL2, PLA2G2A, TICAM2, RPL10, OAS1A, SRP72	2.050714	Cellular component
0042571	Immunoglobulin complex, circulating	3	0.009667	IGHG, IGG-2A, LOC100361105	19.80749	Cellular component
0051434	BH3 domain binding	2	0.052329	BCL2, BAX	37.2067	Molecular function
0005324	Long-chain fatty acid transporter activity	2	0.062463	FABP1, FABP2	31.00559	Molecular function
0015267	Channel activity	2	0.092224	BCL2, BAX	20.67039	Molecular function
0051400	BH domain binding	2	0.092224	BCL2, BAX	20.67039	Molecular function
0048406	Nerve growth factor binding	2	0.092224	A2M, NGFR	20.67039	Molecular function

### Gene Expression in qPCR

**FIGURE 5:**
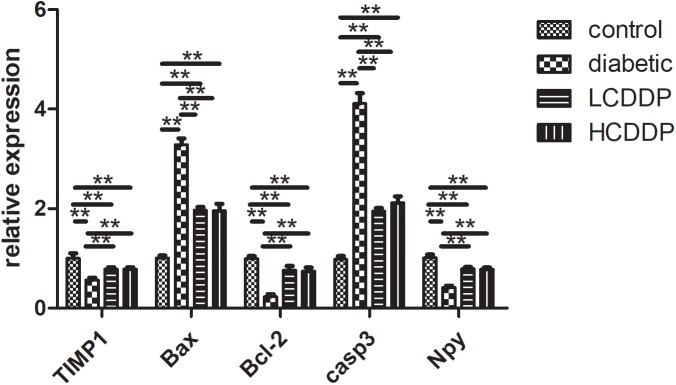
Confirmation of five representative differentially expressed genes by real-time quantitative PCR. LCDDP, low dose Compound Danshen Dripping Pill; HCDDP, high dose Compound Danshen Dripping Pill; Casp3, Caspase 3; Bax, Bcl-2-associated X; NPY, neuropeptide Y. *n* = 6 in each group. ^∗∗^*P* < 0.01.

Real-time quantitative PCR confirmed the results of the gene array. Bax and Casp3 levels increased significantly and TIMP1, Bcl-2, and neuropeptide Y (NPY) levels decreased in diabetic rats (*P* < 0.01, Figure 5). CDDP mediated the expression of these genes in the rat retinas (*P* < 0.01, Figure 5).

### Effect of CDDP on Retina Bcl-2 and Bax Expression in Diabetic Rats

**FIGURE 6:**
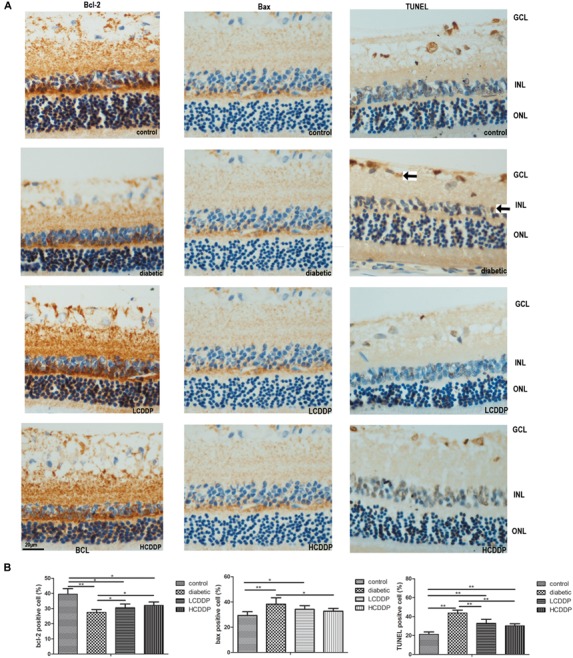
The effect of CDDP on retina Bcl-2 expression, BAX expression and cell apoptosis. **(A)** Images of Bcl-2 immunohistochemistry, Bax immunohistochemistry, and TUNEL analysis. Bcl-2- and Bax-positive cells were localized mainly in the INL. TUNEL-positive cells were localized mainly in the GCL and INL. Arrows show the TUNEL-positive cells. **(B)** Percentages of Bcl-2-, Bax- and TUNEL-positive cells in the retina. LCDDP, low dose Compound Danshen Dripping Pill; HCDDP, high dose of Compound Danshen Dripping pill; GCL, ganglion cell layer; INL, inner nuclear layer; ONL, outer nuclear layer. The results from five sections per eye were averaged. *n* = 6 in each group. ^∗^*P* < 0.05, ^∗∗^*P* < 0.01.

Bcl-2- and Bax-positive cells were localized mainly in the INL. Immunohistochemistry analyses showed that Bcl-2 expression in the retinas of diabetic rats decreased (*P* < 0.01, Figure 6). However, Bax expression increased in the diabetic retinas (*P* < 0.01, Figure 6). After 12 weeks of treatment, Bcl-2 expression increased and Bax expression decreased in both CDDP treatment groups (*P* < 0.05, Figure 6).

### Effect of CDDP on Cell Apoptosis in the Diabetic Rat Retina

TdT-mediated dUTP nick-end labeling-positive cells were localized mainly in the ganglion cell layer (GCL) and INL. TUNEL analysis showed that cell apoptosis in the diabetic rats increased (*P* < 0.01, Figure 6). CDDP treatment reduced cell apoptosis in the diabetic rat retinas (*P* < 0.01, Figure 6).

## Discussion

In this study, we found that high-fat diet and STZ-induced diabetic rats had abnormal b-wave amplitudes and OPs, which indicated that the T2DM rat model had developed DR. Increasing evidence shows that diabetic patients also exhibit similar abnormalities in the early stages of DR (Holopigian et al., 1992; Shirao and Kawasaki, 1998). Interestingly, in our study, we demonstrated for the first time that CDDP had beneficial effects on ERG results in diabetic rats. Without any effect on blood glucose level or body weight, CDDP restored the b-wave amplitude and OPs. These results indicate that CDDP may be an effective drug for treating of DR. Previously, two randomized, double-blind clinical trials showed that CDDP improves the symptoms of non-proliferative DR (NPDR) (Lian et al., 2015; Luo et al., 2015). The current study is the first to provide evidence that CDDP prevents retinal neurodegeneration independent of blood glucose levels in diabetic rats.

Moreover, we found that diabetic rats had thinner ONLs and fewer cells in the ONLs of the retinas. Previously, in other diabetic rodent models, researchers also found similar changes (Barber et al., 1998; Martin et al., 2004). In our study, CDDP significantly improved the thickness of the entire retina and ONL and the cell count in the ONL in diabetic rat retinas. These results indicate that CDDP can delay the progression of DR in a rat model.

To elucidate the mechanism by which CDDP mediates neuroprotection, we performed a whole genome expression array of the retinas in the CDDP-treated and diabetic groups. Our results showed that compared to those in the diabetic group, differentially expressed genes in the CDDP treatment groups were related mainly to the apoptosis pathway. From the TUNEL results, we also observed that the CDDP-treated groups had fewer TUNEL-positive retina cells than the untreated diabetic group. An increasing number of scientists believe that retinal cell apoptosis and reactive gliosis are basic pathological features of early DR (Simo and Hernandez, 2012). Previous studies in human and animal models indicate that DR is associated with apoptosis (Barber et al., 1998; Kern et al., 2000). Taken together, the cell apoptosis-inhibiting property of CDDP might be linked to retina neuroprotection in diabetic rats.

Next step, we searched the gene interaction of differentially expressed genes in the CDDP-treated groups compared to those in the diabetic group. String analysis showed that TIMP1, Bcl-2 and Casp3 were central in the gene interaction network. These genes are involved in apoptosis. Thus, based on the gene expression results, our study also showed that the mechanism of CDDP retinal neuroprotection involves retinal cell apoptosis. First, CDDP prevented the diabetes-induced TIMP1 reduction. TIMP1 and matrix metalloproteinases (MMPs) are important for cell-extracellular matrix (ECM) integrity in the retina (Akahane et al., 2004; German et al., 2008; Hansson et al., 2011). These molecules remodel the ECM to regulate neural organization (Cornelius et al., 1995; Yamada et al., 2001). In addition, TIMP1 was found to inhibit apoptosis in neurons (Ashutosh et al., 2012). A recent clinical trial and animal experiment also suggested that TIMP1 is significantly upregulated in retinal degeneration (Matsuo et al., 1998; Zeiss et al., 1998; Kim et al., 2014). In addition to acting on MMPs (Murphy et al., 2002), TIMP1 also significantly inhibits Bax activity and reduces ONL thinning in the early stages of DR (Kim et al., 2018). Second, in our study, CDDP treatment also inhibited the abnormally high expression of Bax. These results indicate that the neuroprotective effect of CDDP on the diabetic rat retina is mediated by TIMP1 expression inhibition and increased Bax expression, which inhibit apoptosis in the diabetic retina. Bax belongs to the Bcl-family, which plays an important role in mitochondrial apoptosis (Brunelle and Letai, 2009). In contrast, Bcl-2 is an apoptotic cell death blocker (Merry and Korsmeyer, 1997). In our study, both immunohistochemistry and gene expression analyses revealed a significant increase in Bax expression and a reduction in Bcl-2 expression in the diabetic retina. A previous study also showed similar results (Oshitari and Roy, 2005; Kim et al., 2013). Our results showed that CDDP reversed the changes in Bax and Bcl-2 expression. These results indicate that CDDP ameliorated retinal neural cell apoptosis. Caspases are a family of thiol proteases. They are important regulators of the apoptotic cascade and are enzymes induced by high glucose concentrations (Mohr et al., 2002). In present study, we found that Casp3 expression was increased in diabetic rat retinas. These results are consistent with those of previous studies (Gao et al., 2017). We also found that CDDP could inhibit Casp3 expression in the diabetic retina to inhibit apoptosis.

In addition, the present study found that CDDP restored the diminished NPY expression in diabetic rats. Retinal cells express NPY, particularly in neurons (Oh et al., 2002). NPY plays an important role in the development (Prada Oliveira et al., 2003), neuromodulation (D’Angelo and Brecha, 2004), and neuroprotection (Koulu et al., 2004) of the retina. A genome-wide association study (GWAS) in a Finnish population showed that NPY gene polymorphisms are associated with increased incidence of DR (Niskanen et al., 2000; Koulu et al., 2004). The gene can be used as a predictor for earlier DR onset (Jaakkola et al., 2006). Thierry et al. (2015) found that NPY expression was reduced after 3 days of a high-fructose diet in rat models. In an oxygen-induced retinopathy mouse model, researchers also found that NPY expression was decreased (Schmid et al., 2012). Our research provides evidence that CDDP could restore retinal NPY expression in diabetic rats.

## Conclusion

In summary, we found therapeutic properties of CDDP for the prevention of DR. This is the first study to report that CDDP has retinal neuroprotective effects that are independent of blood glucose levels in diabetic rats. These CDDP-induced neuroprotective effects may be mediated by retinal cell apoptosis inhibition and increased NPY expression.

## Author Contributions

XX conceived and designed the experiments. QZ, JZ, TW, and XW performed the experiments. MY, ML, and FP analyzed the data. XX contributed to reagents, materials, and analysis tools. QZ wrote the paper.

## Conflict of Interest Statement

The authors declare that the research was conducted in the absence of any commercial or financial relationships that could be construed as a potential conflict of interest.

## Footnotes

1 http://david.abcc.ncifcrf.gov/

2 https://www.ncbi.nlm.nih.gov/geo/query/acc.cgi?acc=GSE115866
